# Effect of *Bacillus subtilis* DSM 32315 in Diets on Performance and Gut Integrity of Post-Weaning Piglets

**DOI:** 10.3390/ani15131977

**Published:** 2025-07-05

**Authors:** Illa Carla Santos Carvalho, Elenice Andrade Moraes, Débora Cristiane de Oliveira Carvalho, Fabrina de Sousa Luna, Demerson Arruda Sanglard, Afonso Luna Miranda, Isabela Santos Correa, Larissa Tayna Silva Martins, Sara Kauane Brito, Gustavo Roberto Ribeiro Nery, Henrique Gastmann Brand, Guilherme Rocha Moreira, Bruno Alexander Nunes Silva

**Affiliations:** 1 Animal Science Department, Universidade Federal do Vale do São Francisco (UNIVASF), Petrolina 56304-917, PE, Brazil; illlac@hotmail.com (I.C.S.C.); elenice.moraes@univasf.edu.br (E.A.M.); debora.carvalho@univasf.edu.br (D.C.d.O.C.); fabrinaluna@gmail.com (F.d.S.L.); 2 Institute of Agricultural Sciences, Universidade Federal de Minas Gerais (UFMG), Montes Claros 39404-547, MG, Brazil; demerson.ufmg@gmail.com (D.A.S.); afonsolunamiranda@gmail.com (A.L.M.); isabela.correa.ufmg@gmail.com (I.S.C.); larissatayna43a@gmail.com (L.T.S.M.); sarakauane1@gmail.com (S.K.B.); gustavoonery@outlook.pt (G.R.R.N.); 3 Evonik Degussa Brazil Ltda., São Paulo 07222-023, SP, Brazil; henrique.brand@evonik.com; 4 Department of Statistics and Informatics-DEINFO, Universidade Federal Rural de Pernambuco (UFRPE), Recife 52171-900, PE, Brazil; guirocham@gmail.com

**Keywords:** probiotics, intestinal permeability, nursery, pigs

## Abstract

This study evaluated the effect of the replacement of AGPs by a probiotic in diets for piglets in the nursery stage. The dietary treatments were as follows: CON-basal diet (BD); ANT-BD+antibiotic; and PRO-BD+probiotic. From d 35–42 of age, the piglets that received ANT-BD showed a higher average daily weight gain (ADWG) (*p* = 0.0296), followed by those that received PRO and the control. The average daily feed intake (ADFI) was higher (*p* = 0.0224) for PRO- and ANT-fed piglets when compared to CON. From d 43–56, the ADWG was the highest (*p* = 0.0207) in piglets fed ANT. The ADFI was also higher (*p* = 0.0258) in ANT and PRO. Final body weight (BW) was also influenced (*p* = 0.0291), whereas ANT-fed animals, followed by PRO, showed a higher BW compared to CON. For overall nursery performance, PRO showed the highest (*p* = 0.046) ADFI compared to all other treatments. Piglets fed PRO and ANT also showed the highest (*p* = 0.05) end weights. There was no significant difference in the fecal concentration of *Escherichia coli*. Concentrations for the sugar absorption test were higher on days 34 and 49 (*p* < 0.05). The inclusion of *B. subtilis* DSM 32315 does not replace the use of antibiotics with the same level of results but can provide benefits compared to diets without the use of antibiotics.

## Simple Summary

The use of antibiotic growth promoters in post-weaning diets can lead to antibiotic resistance. Probiotics are thought to be a good replacement, improving the gut health of piglets in nursery. The objective of this study was to evaluate the effect of a probiotic, *Bacillus subtilis* DSM 32315, in diets for piglets on their performance and intestinal integrity during the nursery stage. The results indicated that the use of the probiotic significantly influenced performance traits throughout the nursery stage and improved end weights. There was no significant difference in the fecal concentration of *Escherichia coli*, but a higher concentration in the first phase was observed for all treatments. In addition, the sugar absorption test was not influenced by the probiotic or antibiotic but was influenced by the nursery phase. These findings suggest that the inclusion of *Bacillus subtilis* DSM 32315 does not replace the use of antibiotics with the same level of results but can provide several health benefits, improving performance when compared to diets without the use of antibiotics.

## 1. Introduction

In intensive swine farming systems, the most critical period in a piglet’s life is weaning, when animals undergo changes that cause significant stress and can reduce growth rates. In addition, their gastrointestinal tract is still developing at this stage, and their enzyme production is low [1], insufficient for the digestion of protein present in the feed ingredients. The change from highly digestible liquid food to less digestible plant-based feed will influence and modify motility and nutrient absorption in the piglet’s digestive tract, besides the piglet suffering from environmental changes (transfer from farrowing to nursery) and social changes (separation dam and litter regrouping) as well as handling and transportation [2].

All these changes lead to reduced consumption, causing diarrhea and weight loss. To compensate for these effects, it is common practice to add therapeutic alternatives and growth promoters, such as antibiotics, which are mainly used to combat pathogenic microorganisms to reduce this problem. An alternative to antibiotics is the use of probiotics that increase the animal’s immunity, where studies show that these compete with pathogenic bacteria for adhesion to nutrients in the animal’s intestine, suppressing the growth of these undesirable bacteria [3,4].

The excessive use of antibiotics as growth promoters (AGPs) in feed, to maintain the piglet’s health, has raised concerns due to the growing number of resistant antimicrobial agents in animals and humans. In this sense, probiotics have been explored as a feed additive to enhance performance and maintain the intestinal health of pigs in the nursery stage, especially in such a critical period where there is instability in the pig’s microbiota. Probiotics are defined as specific live microorganisms that exert desirable effects and offer benefits to the intestinal health of the host [5]. The establishment of these beneficial bacteria can lead to healthier animals, with this status being achieved more effectively by piglets at weaning with probiotics, so that an environment with desirable bacterial strains can be provided via the diet. The primary mode of action of probiotics involves competitive exclusion against pathogenic microorganisms [6] through adhesion binding on the epithelial surface of the intestine. Competition for nutrients and the production of bacteriocins improve the microbial balance of the host and intestinal health. Therefore, it is necessary to study different strains of probiotics, to further characterize the mechanisms of action and their interaction in the intestinal health of pigs [7] and to comprehend the doses, species and combinations of probiotics appropriate in controlling pathogens to [8] enable a variety of probiotics on the market. The present study aimed to evaluate the effect of replacing AGPs with a probiotic (i.e., *Bacillus subtilis* DSM 32315) in post-weaning diets on performance, intestinal permeability and fecal *E. coli* concentrations.

## 2. Materials and Methods

All methods involving animal handling were performed in accordance with the regulations approved by the Institutional Animal Welfare and Ethics/Protection committee, Universidade Federal de Minas Gerais (UFMG), under the protocol number 296/2017.

### 2.1. Animals and Experimental Design

This study was conducted in the nursery facilities of the swine production farm of the university UFMG/ICA. A total of 54 piglets (27 castrated males and 27 females), TN70 × Talent^®^ (Topigs Norsvin, Den Bosch, The Netherlands), were distributed in a randomized block design according to body weight, sex and litter origin among three dietary treatments with six replications each. The piglets were housed in pens (three animals each) of 1.20 m length × 1.00 m width × 0.80 m height, with a semiautomatic feeder, nipple drinker and plastic slatted floor, with access to feed and water ad libitum. The experimental diets were as follows: a basal diet (BD): CON (negative control); ANT (BD+antibiotics provided during phases 1 and 3 via the addition of 250 g/ton of Lincomycin Hydrochloride (50 mg) + Spectinomycin Sulfate (100 mg)); and PRO (BD+probiotic provided during phases 1, 2, 3 and 4 with the addition 50 g/ton, containing the bacterial strain *Bacillus subtilis* DSM 32315 with least 2 × 10^9^ colony-forming units (CFU) per gram). Whereas DSM stands for Deutsche Sammlung von Mikroorganismen und Zellkulturen (German Collection of Microorganisms and Cell Cultures). This is a well-known microbial culture collection that catalogs and distributes microorganisms and cell cultures for research and development. The “32315” is the specific identifier number assigned to that particular strain of *Bacillus subtilis* within the DSM collection.

The piglets were weaned at 27 d of age with an average body weight of 9.35 ± 1.98 kg and were kept until 73 days of age with an average live weight of 31.41 ± 4.32 kg. The diets (Table 1) were formulated to meet the requirements of each category, following the recommendations of the Brazilian Poultry and Swine Tables [9]. The nutritional program was divided into four phases of development in the nursery: phase 1: from 27 to 34 days; phase 2: from 35 to 42 days; phase 3: from 43 to 56 days; and phase 4: from 57 to 73 days of age. The ambient temperature, relative humidity and photoperiod fluctuated in response to external conditions throughout the day. Overnight, reinforced plastic curtains were used to effectively block the wind and protect the rooms. Each pen was equipped with an individual heating infrared lamp. Ambient temperature and relative humidity were continuously recorded in the nursery, using a data logger (Didai Tecnologia Ltd.a., Campinas, Brazil) placed in the center of the facility at 1 m above the floor. The experimental animals were not challenged. Piglets were vaccinated (Circumvent PCV M) at 26 d of age (1 d prior to weaning) and received a second dose at 42 d of age. The piglets were observed at least three times a day to check for the occurrence of severe diarrhea. During the entire experimental period, only two piglets from the control treatment (CON) were given enrofloxacin once a day for 3 consecutive days at a dose of 0.5 mL/10 kg of body weight.

### 2.2. Measurements and Collected Data

Every morning, feed refusals were collected when available, and fresh feed was immediately distributed once per day between 6:30 am and 7:30 am. Feed intake was determined as the difference between feed allowance and the refusals collected the next morning. The piglets were weighed individually at the beginning and end of each experimental phase. For each phase, the following parameters were calculated: average daily feed intake (ADFI), feed conversion (FC), average daily weight gain (ADWG) and total weight gain (TWG).

### 2.3. Concentration of Escherichia coli in Feces

The feces sampling followed a standard protocol: The animals were monitored, and when they began to defecate, feces were collected. Feces from three periods of collection per phase (first day, middle day and last day) from each individual pig from 6 pens/treatment were sampled and pooled, and an amount of 40 g of feces was separated, marked and frozen (i.e., −80 °C). These samples were then analyzed for *Escherichia coli* presence and concentration using the following method: The samples were aseptically weighed in sterile flasks, homogenized with a buffered aqueous solution and then incubated at 37 °C for 24 h in a 3M™ Petrifilm rapid (3M, Saint Paul, MN, USA) *E. coli* count plate. Decimal dilutions of 10^−1^ were performed in tubes containing 9.0 mL to estimate the bacterial population.

### 2.4. Sugar Absorption Test (SAT)

Six piglets from each treatment were used to conduct the sugar absorption tests (SATs) as markers of intestinal absorption based on methodologies described in [10,11]. One piglet was selected per repetition per treatment, and galactose absorption across the gastrointestinal tract (GIT) using 20% galactose (≥98%; Sigma Aldrich, St Louis, MO, USA) (2.5 mL/kg body weight (BW)) was assessed according to [11]. The galactose SAT was a longitudinal study performed during 3 periods after weaning (34, 49 and 63 d of age). Piglets were fasted for three hours by blocking access to the feeder, though they still had free access to water. An oral dose containing the galactose solution (galactose + distilled water) was then administered via a nasogastric tube. Twenty minutes after administration, a blood sample was taken by venipuncture of the jugular vein. The samples were collected in a lithium-heparin-coated tube and an ethylenediaminetetraacetic acid (EDTA)-coated tube and immediately chilled on ice. The blood was centrifuged for 20 min at 2000× *g* at 4 °C, and aliquots of plasma were stored at −80 °C. Plasma galactose concentrations were then determined using commercial kits (Galactose Colorimetric/Fluorometric Assay Kit—Sigma Aldrich, St Louis, MO, USA) in accordance with the manufacturer’s instructions.

### 2.5. Calculations and Statistical Analyses

During the entire experimental period within the nursery phase, ambient temperature and relative humidity data were analyzed for their maximum and minimum daily averages and variance. The effects of treatments, replicates and live weight were tested according to a general linear analysis of variance (GLM procedure of SAS 9.3 2011). The initial weight of the piglets was considered a covariate in the model. The least-squares procedure (option PDIFF) compared the means when a significant F value was obtained. Piglet performance averages were compared using the Tukey test. For the qualitative and non-parametric data, such as the feces bacterial count, the Mann–Whitney U test was applied. The comparison of means was performed according to the Pdiff option of the SAS procedure using the Tukey test for contrasts. The differences were considered significant if *p* < 0.05. Plasma galactose levels were analyzed using the SAS GLM procedures using the following model:

Yij = μ + TRTi + ej

where μ is the overall average, TRTi is the diet and ej is the residual error. Whenever there was an overall diet effect, differences between two diets were tested using a post hoc Tukey test, maintaining overall type II error, and litter was used as the experimental unit. A GLM was also used to compare differences between days within the diet.

## 3. Results

During the experimental period, the average maximum and minimum temperatures, along with the relative humidity levels, were 25.6 and 23.2 °C, and 77 and 61%, respectively. These values indicate that the animals were kept within their thermal comfort zone since climatic requirements in the rooms were met.

### 3.1. Performance

During the period from 27 to 34 d, the diets did not influence (*p* > 0.10; Table 2) any of the evaluated traits. Between 35 and 42 days, an effect of the antibiotic was observed in the 27-to-34-day phase, as piglets receiving the antibiotic diet showed a greater daily weight gain, ADWG (*p* = 0.0296), followed by those receiving *B. subtilis* DSM 32315 and the control (553 vs. 505 vs. 450 g/d, respectively). The ADFI was higher (*p* = 0.0224) for *B. subtilis* DSM 32315- and antibiotic-fed piglets (636 vs. 611 g/d) compared to the control. During the period from 43 to 56 d, the ADWG was the highest (*p* = 0.0207) in piglets treated with antibiotics (599 g/d). Moreover, the ADFI was also higher in piglets treated with ANT and PRO. As a consequence, the final BW was also influenced (*p* = 0.0291), whereas ANT-fed animals, followed by PRO, showed a higher BW compared to the control-fed group (22.71 vs. 21.39 vs. 20.39 kg, respectively). During the last period from 57 to 73 d, the highest (*p* = 0.0097) ADFI was observed in piglets treated with PRO when compared to the other two treatments. All other parameters were not influenced (*p* > 0.10). As for overall performance, piglets fed PRO showed the highest (*p* = 0.046) ADFI compared to all other diets (on average 842 vs. 810 g/d, respectively, for PRO and ANT). As a consequence, piglets fed PRO and ANT showed the highest (*p* = 0.050) end weights (22.47 vs. 22.45 vs. 21.17 kg, respectively, for PRO, ANT and CON).

### 3.2. Intestinal Permeability

**Figure 1:**
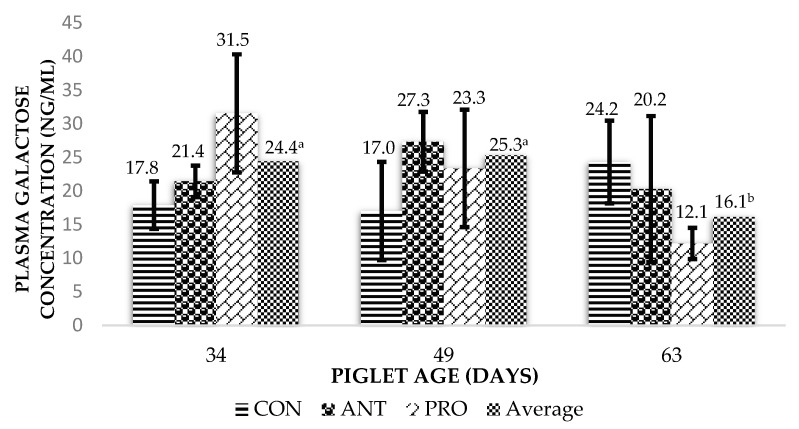
Intestinal integrity of piglets treated or not treated with an antibiotic or probiotic at different ages after weaning (LS means). Means followed by different letters differ from each other according to Tukey’s test (*p* < 0.05). CON, BD-negative control; ANT, BD+antibiotic (Lincomycin Hydrochloride + Spectinomycin Sulfate); PRO, BD+probiotic (*Bacillus subtilis* DSM 32315).

As shown in Figure 1, no effect was observed between diets and their interactions regarding the intestinal integrity of piglets when considering the galactose absorption capacity (*p* > 0.10). Nevertheless, on d 34 and 49 of age, plasma galactose concentrations were higher (*p* < 0.05) for all treatments when compared to d 63 of age.

### 3.3. Concentration of Escherichia coli in Feces

There were no significant (*p* > 0.10; Table 3) effects of treatments on *Escherichia coli* concentrations in all four phases evaluated. In phase 2, none of the treatments showed *Escherichia coli* growth in direct dilution readings.

## 4. Discussion

At weaning, piglets are challenged by several factors (i.e., environmental and nutritional), which can impact and lead to imbalanced intestinal micro-ecology, intestinal dysfunction, decreased nutrient absorption and subsequently a low growth rate [12]. With the global trend of banning the use of antibiotics as growth promotors, the use of probiotics has become an alternative to promote benefits and improve animal intestinal health [12,13,14]. Our study confirms the results of previous studies reporting positive effects of dietary supplementation with *B. subtilis* DSM 32315 on growth performance in weanling piglets, wherein supplementation of *B. subtilis* DSM 32315 may exert beneficial effects. However, the literature on the effect of *B. subtilis* DSM 32315 supplementation as a replacement for the use of antibiotics in piglets is not fully homogeneous, likely because of different experimental designs.

Growth performance may be influenced by the dynamic interplay between the gut microbiota and intestinal function [15]. Probiotic treatments can positively impact the intestinal ecosystem, potentially leading to improved nutrient absorption and overall health. From 35 to 42 d of age, piglets that received the diets containing ANT or PRO showed an improved ADWG and ADFI, demonstrating superior performance compared to piglets in the control group. Improvement related to the use of antibiotics is well documented in the literature and is related to a mechanism of action that reduces the concentration of pathogenic bacteria which impact gut integrity, nutrient absorption and immune modulation [16]. As for the use of probiotics, these can improve piglet growth performance by improving gut health, nutrient absorption and immune function. The use of probiotics can reduce the concentration of harmful bacteria in the intestine [17], which will lead to an improvement in intestinal barrier function and nutrient absorption efficiency together with the establishment of a more favorable gut microbial environment through the expansion of beneficial bacteria and the suppression of harmful strains such as *Escherichia coli*, *Salmonella* and *Clostridium perfringens* [18,19,20]. Our findings are in agreement with those of the authors of [12], who observed that the use of *B. subtillis DSM 32315* is capable of improving the growth performance of piglets. Also in agreement, the authors of Ref. [19], working with piglets in two different growth phases, from 0 to 14 and 15 to 42 days of age, using the same strain as in our study, observed a higher average daily weight gain (+20 and +44 g/d, respectively) compared to the control-fed group. In piglets that do not yet have a stabilized microbial population and are still subjected to the stress caused by weaning, the intestine will need more time to adapt to the new situation. These findings altogether demonstrate that the use of *B. subtillis* DSM 32315 can be an alternative to maintain performance and replace the use of antibiotics in post-weaning piglet diets.

Although intestinal permeability, studied via the sugar absorption test, was not influenced by the treatments when compared within the three evaluated stages, still, a higher numerical difference was seen for the ANT- and PRO-fed piglets when compared to the control on d 34 and 49. One could infer that the improved voluntary feed intake and growth rate observed during this period could be associated with better intestinal integrity. According to [21,22,23], intestinal permeability is correlated with the height of the animal’s villi, the site of the absorption of nutrients that would be used by the animal, influencing its development. In this sense, better gut integrity would allow better efficiency for nutrient absorption and therefore improved growth rates.

Intestinal permeability was influenced by the age of the piglets. The galactose absorption test is an indicator that measures transepithelial permeability, thus providing data on the integrity and function of the intestinal barrier [10,11,24]. On d 34 and 49 of age, piglets showed a higher value for galactose absorption when compared to d 63. A possible explanation would be that the transition from more elaborate diets (i.e., phase 1, phase 2 and phase 3) to a less elaborate diet (i.e., phase 4) with higher levels of soybean meal inclusion could have compromised intestinal health and integrity in all treatments, as it is well known that soybean meal has high levels of allergenic (i.e., the proteins glycinin and β-conglycinin) and antinutritional factors (i.e., trypsin inhibitors, oligosaccharides, lectins and antigens) that can impact gut integrity [25,26].

No difference in *Escherichia coli* concentration was observed among treatments throughout the study, which may be associated with the dosage used of *B. subtilis* DSM 32315, which may not have been sufficient to counteract the bacteria. In a previous study [27], high doses of a probiotic (*B. subtilis* GCB-13-001 1 × 10^9^ CFU/kg) showed a better effect on the gastrointestinal microbiota in weaned piglets, reducing the *E. coli* count. Nevertheless, a numerical reduction in the concentration according to the phases was observed, wherein a higher concentration of *Escherichia coli* was seen in the early phase when compared to the later phases. The population of coliforms and *E. coli*, a bacterium responsible for post-weaning diarrhea, increases in the host’s intestine after weaning [28]. Our findings are comparable to those of previous studies, such as [29], which evaluated the use of *Bacillus subtilis* B27 (2.0 × 10^8^ CFU/kg) and reported that its inclusion did not affect fecal *Escherichia coli* counts. Alternatively, Ref. [30] showed positive results using probiotics which, according to the authors, is considered an indicator of better intestinal health in animals, wherein piglets were supplemented with *Bacillus subtilis* H4 (6 × 10^11^ CFU/mL) and *E. coli* counts gradually decreased in pigs fed the diet containing probiotics.

## 5. Conclusions

In conclusion, our findings suggest that the inclusion of *Bacillus subtilis* DSM 32315 does not replace the use of antibiotics with the same level of results but can provide several health benefits, improving performance when compared to diets without the use of antibiotics.

## Figures and Tables

**Table 1:** Ingredients and nutrient composition of the basal diets.

	Phases *			
Ingredients	1	2	3	4
Corn	31.490	37.660	48.130	59.004
Soybean meal	20.000	22.000	26.000	28.000
Pre-cooked corn	10.000	10.000	7.000	0
Spray-dried blood plasma	5.000	3.000	0	0
Bakery by-product meal	5.000	5.000	5.000	5.000
Soybean oil	3.550	3.350	3.400	3.800
Milk whey	21.000	15.000	6.000	0
Calcium phosphate	1.694	1.901	2.090	2.025
Calcium carbonate	0	0	0.106	0.265
Sodium chloride	0.400	0.400	0.400	0.400
Flavoring additive ^1^	0.030	0.030	0.030	0.030
Organic acid blend ^2^	0.200	0.000	0.000	0.000
L-Lysine	0.415	0.475	0.587	0.511
DL-Methionine	0.240	0.237	0.270	0.200
L-Threonine	0.159	0.188	0.281	0.239
L-Tryptophan	0.092	0.096	0.090	0.067
L-Valine	0.100	0.120	0.166	0.110
Mineral supplement ^3^	0.100	0.100	0.100	0.100
Vitamin premix ^4^	0.050	0.050	0.050	0.050
Micotoxin deactivator ^5^	0.100	0.100	0.100	0.100
Zinc oxide 80%	0.280	0.240	0.200	0.100
Nutritional Specifications—Nutritional Composition				
Metabolizable energy (kcal/kg)	3.435	3.412	3.377	3.367
CP (%)	19.48	18.76	18.04	18.27
Calcium (%)	0.683	0.710	0.715	0.720
Digestible phosphorus (%)	0.439	0.430	0.400	0.360
SID lysine	1.35	1.30	1.26	1.20
SID methionine + cystine	0.81	0.78	0.78	0.72
SID threonine	0.88	0.84	0.84	0.80
SID valine	0.95	0.91	0.88	0.84
SID tryptophan	0.30	0.29	0.25	0.23

* Phase 1: from 27 to 34 days; phase 2: from 35 to 42 days; phase 3: from 43 to 56 days; and phase 4: from 57 to 73 days of age. ^1^ Food sweetener. ^2^ Mixture of organic acids: formic acid, acetic acid, propionic acid, lactic acid, citric acid, carrier and anti-caking agent. ^3^ Carbo-amino-phospho chelate of cobalt (cobalt: 102 mg/kg), Carbo-amino-phospho chelate of copper (copper: 7500.00 mg/kg), Carbo-amino-phospho chelate of chromium (chromium 100.00 mg/kg), Carbo-amino-phospho chelate of iron (iron: 52.00 g/kg), Carbo-amino-phospho chelate of manganese (manganese 23.00 g/kg), Carbo-amino-phospho chelate of selenium (selenium 184.00 mg/kg), Zinc Carboamino-phosphochelate (Zinc 57.50 g/kg), Butylated Toluene Hydroxide (BTH) and Calcium Iodide (Iodine 665 mg/kg). ^4^ Guaranteed levels per kg of feed: Vitamin A (11,250 IU/kg), Vitamin D3 (1900 IU/kg), Vitamin E (100 IU/kg), Vitamin K (5 mg/kg), Biotin (0.5 mg/kg), Folic Acid (4.5 mg/kg), Niacin (60 mg/kg), Pantothenic Acid (30 mg/kg), Vitamin B2 (1000 mg/kg), Vitamin B1 (4 mg/kg), Vitamin B6 (6 mg/kg) and Vitamin B12 (50 mcg/kg). ^5^ Mycotoxin binder. Source: own authorship (2021); SID: standardized ileal digestibility.

**Table 2:** The effect of probiotic and antibiotic supplementation in the diet on the growth performance of piglets during the nursery phase (LS means).

Treatments					
Parameters	CON	ANT	PRO	RSD	*p*-Value
Phase 1 (27 to 34 days)					
Initial BW, kg	9.29	9.29	9.30	0.61	0.7468
Final BW, kg	10.44	11.05	10.96	1.21	0.5427
ADWG, g/d	164	251	236	10	0.0789
ADFI, g/d	277	308	310	4	0.4863
FC	1.68	1.23	1.31	1.12	0.0605
Phase 2 (35 to 42 days)					
Final BW, kg	13.59	14.92	14.47	4.04	0.1450
ADWG, g/d	450 ^c^	553 ^a^	505 ^b^	10	0.0296
ADFI, g/d	539 ^b^	611 ^a^	636 ^a^	10	0.0224
FC	1.20	1.11	1.26	0.13	0.2358
Phase 3 (43 to 56 days)					
Final BW, kg	20.39 ^c^	22.71 ^a^	21.39 ^b^	8.22	0.0291
ADWG, g/d	523 ^b^	599 ^a^	532 ^b^	10	0.0207
ADFI, g/d	784 ^b^	845 ^a^	837 ^a^	5	0.0258
FC	1.49	1.41	1.57	0.42	0.1388
Phase 4 (57 to 73 days)					
Final BW, kg	30.46	31.74	32.02	19.91	0.5554
ADWG, g/d	630	565	664	14	0.4110
ADFI, g/d	1.125 ^b^	1.089 ^b^	1.169 ^a^	49	0.0097
FC	1.78	1.93	1.76	1.27	0.6273
Total period					
TWG, kg	21.17 ^b^	22.45 ^a^	22.47 ^a^	1.41	0.0503
ADWG, g/d	492	522	506	4	0.4415
ADFI, g/d	778 ^b^	810 ^b^	842 ^a^	74	0.0460
FC	1.58	1.55	1.66	0.04	0.7862

BW: body weight; ADWG: average daily weight gain; ADFI: average daily feed intake; FC: feed conversion; TWG: total weight gain. Means followed by different letters in the same line differ from each other according to the Tukey’s test (*p* < 0.05). CON, BD-negative control; ANT, BD+antibiotic (Lincomycin Hydrochloride + Spectinomycin Sulfate); PRO, BD+probiotic (*Bacillus subtilis* DSM 32315). RSD = residual standard deviation.

**Table 3:** Effect of probiotic and antibiotic supplementation in diet on fecal *Escherichia coli* concentrations in piglets during nursery phase (LS means).

Period	CON	ANT	PRO	*p*-Value
27 to 34 days	4.12 × 10^5^	3.33 × 10^6^	9.48 × 10^5^	0.208
35 to 42 days	0	0	0	0.406
43 to 56 days	2.60 × 10^4^	1.74 × 10^5^	3.30 × 10^5^	0.900
57 to 73 days	0	9.75 × 10^3^	3.03 × 10^3^	0.700

CON, BD-negative control; ANT, BD+antibiotic (Lincomycin Hydrochloride + Spectinomycin Sulfate); PRO, BD+probiotic (*Bacillus subtilis* DSM 32315). *Escherichia coli*—CFU/gram of feces.

## Data Availability

The raw data supporting the conclusions of this article will be made available by the authors on request.
